# Role of Diffusion Tensor Imaging in Early Diagnosis and Characterization of Movement Disorders

**DOI:** 10.7759/cureus.53580

**Published:** 2024-02-04

**Authors:** M Meyyappan, Biji Babu, M Anitha, Gopinath Ganesan, Anita S, Paarthipan Natarajan

**Affiliations:** 1 Radiology, Panimalar Medical College Hospital and Research Institute, Chennai, IND; 2 Radiodiagnosis, Saveetha Medical College and Hospital, Chennai, IND

**Keywords:** early diagnosis, atypical parkinson’s disease, essential tremors, parkinson’s disease, movement disorders, diffusion tensor imaging

## Abstract

Background: Symptoms of movement disorders in early stages are similar, which makes definite diagnosis difficult. Hence this study was conducted to explore the role of diffusion tensor imaging (DTI) in enhancing the early diagnosis and characterization of movement disorders.

Methodology: A cross-sectional study was conducted including 60 subjects. All of them were reviewed using conventional magnetic resonance imaging (MRI) and movement disorder DTI protocol. Commercially available software was used to produce fractional anisotropy (FA) maps. Post-processing 3D reconstruction was done to obtain tractograms. Both single and multiple regions of interest (ROIs) were selected for tractography in the pons, midbrain, substantia nigra (SN) and cerebellum. MRI and DTI images were interpreted and correlated with confirmatory diagnosis.

Results: According to DTI diagnosis, out of the 30 cases, 28 had movement disorders. Among cases, 36.67% had Parkinson’s disease (PD), 23.33% had progressive supranuclear palsy (PSP), 16.67% had essential tremor, 13.33% had multi-system atrophy (MSA) C, and 3.33% had MSA P. DTI correctly classified all cases with PD and PSP. All cases with long disease duration and 88.24% of cases with short disease duration were also correctly classified. A statistically significant difference was observed in the proportion of diagnosis between DTI and conventional MRI.

Conclusion: DTI has high sensitivity and specificity for the diagnosis of movement disorders. It is capable of early diagnosis of movement disorders and also differentiating and subcategorizing them.

## Introduction

Movement disorders are neurologic syndromes in which there is either an excess of movements or a paucity of voluntary and automatic movements, unrelated to weakness or spasticity [1]. Idiopathic Parkinson’s disease (PD) and atypical parkinsonian disorders (APD), comprising multi-system atrophy (MSA), progressive supranuclear palsy (PSP), and corticobasal degeneration (CBD) are included in idiopathic parkinsonian movement disorders, which are a group of distinct neurodegenerative diseases.

It has been studied that movement disorders are highly prevalent in the community globally. In India, movement disorders constitute 3-8% of neurological disorders. The crude prevalence rate (CPR) varies from 31 to 45/100,000 in adults above 60 years of age. The frequency is higher in rural India due to poor awareness regarding diagnosis and treatment options and lack of health facilities. These disorders are also subject to substantial under-recognition and under-treatment [2,3].

PD, MSA, essential tremor, and PSP have similar symptoms in the early stages due to which definite diagnosis becomes difficult. Overlapping of clinical features can cause misdiagnosis in early cases [4]. In the early stages of the disease, atypical PD comprises 20% of the patients presenting with Parkinsonism [5,6].

Conventionally, movement disorders are diagnosed using basic magnetic resonance (MR) sequences such as susceptibility-weighted image (SWI), T2W, and proton density SE sequences. However, conventional magnetic resonance imaging (MRI) is limited by many factors, including the fact that the features manifest late and for the differential diagnosis their specificity and sensitivity are low. These methods often cannot capture the subtle microstructural changes within the white matter tracts that may precede observable clinical symptoms [7].

Diffusion tensor imaging (DTI) is an advanced and sophisticated MR technique, which along with conventional MRI imaging helps in visualizing and assessing the fiber tract pathways. It can also help visualize the diffusion of white matter in a 3D presentation in the direction of fibers (axons). DTI and diffusion tensor tractography (DTT) provide for detailed imaging of the cerebellum's major afferent and efferent circuits as well as their links to the brainstem [8]. This combined with structural MR imaging can help detect impairment in extrapyramidal circuits, especially in patients suffering from movement disorders in the early stages of disease progression.

In parkinsonian disorders, DTI can become a promising tool when used in combination with DTT to differentially diagnose and evaluate the neurodegeneration of specific pathways. DTI can also play a role in identifying and differentiating involved areas in various subtypes of MSA, PSP, and essential tremor [9]. This will not only facilitate early diagnosis but also provide a window of opportunity for early intervention, refine diagnostic criteria, and tailor the treatment strategies based on specific underlying pathology.

Hence this study was conducted to explore the role of DTI in enhancing the early diagnosis and characterization of movement disorders, with regards to PD, essential tremors, and atypical PD including MSA and PSP. The study also aimed to differentiate the different movement disorders with the help of DTI, to assess if DTI has a role in earlier diagnosis of movement disorders when compared to conventional MR imaging, and to assess if DTI can make the diagnosis of a movement disorder when the conventional imaging is normal.

## Materials and methods

A cross-sectional study for a duration of 12 months was conducted under the Department of Radio Diagnosis at a tertiary care hospital in Chennai. Patients referred to the Radiology Department with clinical suspicion of various movement disorders from the Neurology Department were included in the study as cases. This number was 30. An equal number of controls were also included. Controls included healthy volunteers. The total sample size was 60. The convenience sampling technique was used as cases were only available in the hospital, and the lack of early diagnosis made it difficult to identify these cases in community settings. Inclusion criteria were patients of any gender with clinically proven or highly suspected movement disorders of age 18 years and above. They were diagnosed clinically in the Neurology Department. Exclusion criteria were those patients with contraindications for MRI (including claustrophobia), patients with advanced movement disorders who were non-compliant for the MR examination, and all those patients who did not give consent to be a part of the study.

The institutional ethical committee was approached for ethical approval before commencing the study. Patients were briefed and written informed consent was obtained from them after explaining the procedure. Utmost care was taken to maintain the privacy and confidentiality of the patients. For the 60 subjects who were reviewed using conventional MRI, the sequencing for the brain included T1WI sagittal, T2WI axial, DWI axial, FLAIR axial, and coronal and Fast Field Echo (FFE) sequences. These sequences took approximately 15 minutes to be recorded. Following conventional MRI sequences, all subjects were subjected to a proposed set of sequences. This was called the movement disorder DTI protocol, which included a 3D T1WI (approximate time for the sequence: 5 minutes) and DTI is media® sequence (approximate time for the sequence: 4 minutes).

The total time required for the study was approximately 25 minutes. All images were transferred and viewed in a dedicated MRI workstation where image reconstruction and post-processing analysis were performed. The post-processing of the acquired DTI images was subsequently performed by the use of commercially available software to produce FA (fractional anisotropy) maps. Using post-processing 3D reconstruction, a tractogram was obtained. Greyscale and colored FA maps were produced in every case. The red color was used to code for left to right direction, blue for craniocaudal, and green for dorsal to dorsal. Both single and multiple areas of interest (ROI) in the pons, midbrain, substantia nigra (SN), and cerebellum were chosen for tractography using the same post-processing methods. Using a threshold of FA less than 0.2, the tractography color method was designed to display the major anisotropy and direction of the tracts. ROI was placed in color-coded FA maps superimposed over 3D T1WI and FA value was recorded in SN, middle cerebellar peduncle (MCP), transverse pontine fibers, superior cerebellar peduncle (SCP), and dentate nucleus in all subjects.

ROI placement

ROI in SN was drawn using the following method. The diameter of each ROI was one voxel. First step: The B0 image was used to identify the prominently located red nucleus, subthalamic nucleus, and SN. Since much of the SN lies below the red nucleus, one slice that is inferior to this was taken into consideration. The SN was consistently evident in this inferior slice, but the red nucleus was either barely visible or had disappeared entirely. The ventral SN was the main focus of this technique. Ensuring that the ROI remained inside the brainstem, the first ROI was inserted in the hypointense portion of the rostral segment of the SN. To avoid creating an overlap between the two circles, the middle ROI was positioned lateral and posterior to the first circle. Ultimately, the caudal ROI was positioned so that it was posterior and lateral to the central circle, preventing the two circles from overlapping. The FA map and color map were utilized to ensure that none of the three ROIs were placed laterally in the cerebral peduncle. For the statistical analysis, the mean value from the left and right ROI in the SN was utilized. Only FA value in caudal ROI was documented as it is considered highly specific [10]. Rostral and middle ROI values were used to confirm the presence of lateral to medial gradient. ROI in SCP was placed lateral to SCP decussation in the axial plane. ROI in MCP was placed lateral to the fourth ventricle at the level of the pons. ROI in transverse pontine fiber (TPF) was placed posterior to the pyramidal tract in the axial plane. ROI in dentate the nucleus was placed by identification of the dentate nucleus in SWI sequence, seen as an undulating semicircular structure on either side of the midline in the cerebellum.

The mean of FA was determined for each corresponding left and right ROI. FA values were compared with previously available normal values. The normal FA value in the caudal region of SN was taken as above 0.6 [11]. The normal FA value in MCP was taken as 0.81+/-0.07. The normal FA value in TPF was taken as 0.69+/-0.04 [11]. The normal FA value in SCP was taken as 0.76+/-0.04. The normal FA value in the dentate nucleus was taken as 0.32-0.58 [12]. DTI diagnosis was done based on the FA reduction at specific sites as described below. If FA is reduced in the caudal region of SN, the diagnosis is PD [10]. If FA is reduced in MCP and TPF, the diagnosis is MSA C. If FA is reduced in MCP alone, the diagnosis is MSA P. If FA is reduced in SCP alone, diagnosis is PSP [12]. If FA is reduced in dentate and SCP, the diagnosis is essential tremor [12]. Fiber tract involvement and fiber tract color characteristics (normal or reduction or lost) were noted. The subjects were interpreted by two senior radiologists. One radiologist interpreted conventional MRI and another radiologist interpreted sequences in movement disorder DTI protocol. Radiologist reporting conventional MRI was blinded from both clinical and DTI diagnosis. Similarly, radiologist reporting DTI was blinded from both clinical and conventional MRI diagnoses. Clinical diagnosis was the confirmatory diagnosis when conventional MRI was normal. Conventional MRI was considered the confirmatory diagnosis when it showed abnormal findings. In the end, the conventional MRI diagnosis and DTI diagnosis were compared with each other and both these diagnoses were also independently compared with the confirmatory diagnosis.

Statistical analysis

Descriptive analysis was carried out by frequency and percentage for categorical variables. Continuous variables were presented as mean ± SD. An independent t-test was used to compare the mean ± SD of continuous variables between the two groups. The diagnostic accuracy of DTI diagnosis in correctly identifying the disorders was assessed by calculating sensitivity, specificity, positive predictive value (PPV), negative predictive value (NPV), accuracy, and area under curve (AUC). The marginal homogeneity test was used to compare paired categorical variables with more than two categories. P-value <0.05 was considered statistically significant. Data was analyzed by using coGudie software, V.2 [13].

## Results

A total of 30 cases (i.e., with movement disorder) and 30 controls (i.e., without movement disorder) were included in the study. The mean (±SD) age was 58.57 (±13.22) years among cases and 53.37 (±12.02) years among controls; the mean difference was statistically insignificant (p=0.116). The male:female ratio was 21:9 among both cases and controls.

According to DTI diagnosis among 60 subjects, 28 had movement disorder and 32 had no movement disorder. The sensitivity, specificity, PPV, and NPV of DTI diagnosis in correctly predicting movement disorder were 93.33%, 100%, 100%, and 93.75%, respectively (Table 1).

**Table 1:** Diagnostic accuracy of DTI diagnosis in predicting movement disorder CI, confidence interval; DTI, diffusion tensor imaging; PPI, positive predictive value; AUC, area under curve; NPV, negative predictive value

Parameters	Values	95% CI lower	95% CI upper
Sensitivity	93.33%	77.93%	99.18%
Specificity	100%	88.43%	100%
PPV	100%	87.66%	100%
NPV	93.75%	79.19%	99.23%
Accuracy	96.67%	88.47%	99.59%
AUC	0.967	0.921	1

Among the cases, as per DTI diagnosis, 36.67% (n=11) had PD, 23.33% (n=7) had PSP, 16.67% (n=5) had essential tremor, 13.33% (n=4) had MSA C, 3.33% (n=1) had MSA P, and 6.67% (n=2) were normal. Confirmatory diagnosis was considered as clinical diagnosis when conventional MRI was normal whereas conventional MRI was considered confirmatory when conventional MRI was abnormal. According to confirmatory diagnosis, 36.67% (n=11) had PD, 23.33% (n=7) had PSP, 20% (n=6) had essential tremor, and 20% (n=6) had MSA. Among six cases confirmed as MSA, three were diagnosed as MSA by clinical diagnosis whereas two were diagnosed as MSA C, and one was diagnosed as MSA P according to conventional MRI (Table 2).

**Table 2:** Diagnosis among cases (N=30) DTI, diffusion tensor imaging; PD, Parkinson’s disease; PSP, progressive supranuclear palsy; MSA, multi-system atrophy; MRI, magnetic resonance imaging

Characteristics	Frequency (%)
DTI diagnosis	
PD	11 (36.67%)
PSP	7 (23.33%)
Essential tremor	5 (16.67%)
MSA C	4 (13.33%)
MSA P	1 (3.33%)
Normal	2 (6.67%)
Conventional MRI diagnosis	
PD	6 (20%)
PSP	4 (13.33%)
MSA C	2 (6.67%)
MSA P	1 (3.33%)
Normal	17 (56.67%)
Clinical diagnosis	
PD	11 (36.67%)
PSP	3 (10%)
Essential tremor	6 (20%)
MSA	4 (13.33%)
Atypical PD	6 (20%)
Confirmatory diagnosis	
PD	11 (36.67%)
PSP	7 (23.33%)
Essential tremor	6 (20%)
MSA	6 (20%)

DTI diagnosis correctly classified all the cases with PD and PSP whereas there was one misclassification of essential tremor as normal and one misclassification of MSA as normal (Table 3).

**Table 3:** Diagnostic accuracy of DTI diagnosis in correctly classifying different disorders CI, confidence interval; PD, Parkinson’s disease; PSP, progressive supranuclear palsy; MSA, multi-system atrophy; PNP, positive predictive value; NPN, negative predictive value; AUC, area under curve

Parameters	PD			PSP			Essential tremor			MSA		
	Values	95% CI lower	95% CI upper	Values	95% CI lower	95% CI upper	Values	95% CI lower	95% CI upper	Values	95% CI lower	95%CI upper
Sensitivity	100%	71.51%	100%	100%	59.04%	100%	83.33%	35.88%	99.58%	83.33%	35.88%	99.58%
Specificity	100%	82.35%	100%	100%	85.18%	100%	100%	85.75%	100%	100%	85.75%	100%
PPV	100%	71.51%	100%	100%	59.04%	100%	100%	47.82%	100%	100%	47.82%	100%
NPV	100%	82.35%	100%	100%	85.18%	100%	96%	79.65%	99.90%	96%	79.65%	99.90%
Accuracy	100%	82.35%	100%	100%	88.43%	100%	96.67%	82.78%	99.92%	96.67%	82.78%	99.92%
AUC	1	1	1	1	1	1	0.917	0.753	1	0.917	0.753	1

**Figure 1:**
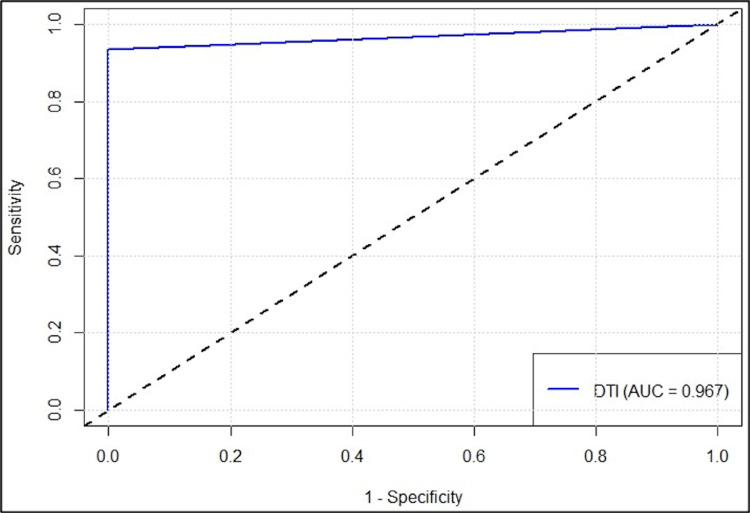
ROC curve of DTI diagnosis in predicting movement disorder DTI, diffusion tensor imaging; AUC, area under curve

43.33% of the cases (n=13) had long disease duration and 56.67% (n=17) had short disease duration. 13/13 (100%) of the cases with long disease duration were correctly classified by DTI diagnosis whereas 15/17 (88.24%) of the cases with short disease duration were correctly classified by DTI diagnosis (Figure 1). 

Among cases diagnosed by conventional MRI as PD (n=6), MSA C (n=2), MSA P (n=1), and PSP (n=4), it was the same according to DTI diagnosis as well. However, among 17 cases diagnosed by conventional MRI as normal, only 11.76% (n=2) were diagnosed as normal as per DTI diagnosis whereas 29.41% (n=5) were diagnosed as PD, 11.76% (n=2) were diagnosed as MSA C, 17.65% (n=3) were diagnosed as PSP, and 29.41% (n=5) were diagnosed as essential tremor. Hence, there was a statistically significant difference in the proportion of diagnosis between DTI and conventional MRI (p=0.005) (Table 4).

**Table 4:** Comparison of DTI diagnosis and conventional MRI diagnosis among cases (N=30) DTI, diffusion tensor imaging; PD, Parkinson’s disease; PSP, progressive supranuclear palsy; MSA, multi-system atrophy; MRI, magnetic resonance imaging

DTI diagnosis	Conventional MRI diagnosis						P-value
	PD (n=6)	MSA C (n=2)	MSA P (n=1)	PSP (n=4)	Essential tremor (n=0)	Normal (n=17)	
PD	6 (100%)	0 (0%)	0 (0%)	0 (0%)	0 (0%)	5 (29.41%)	0.005
MSA C	0 (0%)	2 (100%)	0 (0%)	0 (0%)	0 (0%)	2 (11.76%)	
MSA P	0 (0%)	0 (0%)	1 (100%)	0 (0%)	0 (0%)	0 (0%)	
PSP	0 (0%)	0 (0%)	0 (0%)	4 (100%)	0 (0%)	3 (17.65%)	
Essential tremor	0 (0%)	0 (0%)	0 (0%)	0 (0%)	0 (0%)	5 (29.41%)	
Normal	0 (0%)	0 (0%)	0 (0%)	0 (0%)	0 (0%)	2 (11.76%)	

Among clinically diagnosed cases as PD (n=11) and PSP (n=3), it was the same according to DTI diagnosis as well. However, among six cases clinically diagnosed as atypical PD, 33.33% (n=2) were diagnosed as MSA C as per DTI diagnosis whereas 66.67% (n=4) were diagnosed as PSP. Among four cases clinically diagnosed as MSA, DTI diagnosis was able to subcategorize 50% (n=2) as MSA C and 25% (n=1) as MSA P whereas 25% (n=1) was diagnosed as normal. Among six cases clinically diagnosed as essential tremors, 83.33% (n=5) were diagnosed as essential tremors as per DTI diagnosis whereas 16.67% (n=1) were diagnosed as normal.

**Figure 2:**
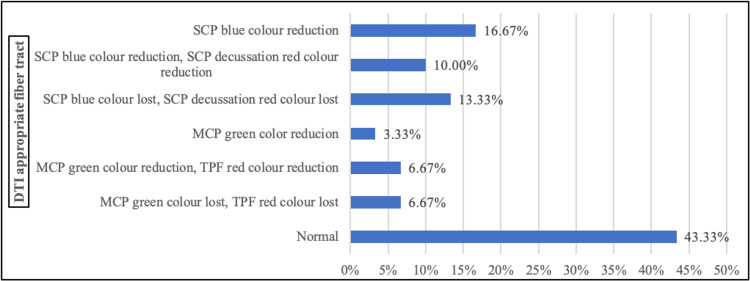
DTI appropriate fiber tract color among cases (N=30) SCP, superior cerebellar peduncle; TPF, transverse pontine fiber; MCP, middle cerebellar peduncle; DTI, diffusion tensor imaging

Among 30 cases, fiber tract was normal in 43.33% (n=13), reduction of SCP blue color was present in 16.67% (n=5), reduction of SCP blue color along with the reduction of SCP decussation red color was present in 10% (n=3), loss of SCP blue color along with loss of SCP decussation red color was seen in 13.33% (n=4), reduction of MCP green color was seen in 3.33% (n=1), reduction of MCP green color along with reduction of TPF red color was present in 6.67% (n=2), and loss of MCP green color along with loss of TPF red color was seen in 6.67% (n=2) (Figure 2).

## Discussion

In the present study, 30 cases and equal controls were assessed for movement disorders using conventional MRI with DTI, to explore the role of DTI in enhancing the early diagnosis and characterization of movement disorders, with regard to PD, essential tremors, and atypical PD including MSA and PSP. It was observed that the number of males in the study exceeded the number of females. This result coincided with other studies conducted by Vaillancourt DE et al., and Reimão S et al., that used high-resolution DTI to detect and distinguish de novo patients with PD at an early stage from healthy individuals [10,12].

Using DTI, two further earlier studies by Michel et al. and Braak et al. demonstrated that PD patients had lower FA in SN when compared to healthy persons [14,15]. The individuals in this study were not as seriously handicapped as those in these trials. Both short- and long-term instances were included in the current investigation, which revealed differences in FA values between the two types of cases that had not before been shown. Additionally, this work adds to the body of prior research by demonstrating that early-stage, de novo PD patients had lower FA in the SN.

According to some research, FA measurements might not reliably identify PD patients. For example, an AUC (0.653) in research by Hodaie et al. was deemed low, indicating that DTI might not be a promising technique for detecting PD in individual people [16]. In this investigation, the PPV of DTI diagnosis was higher than the NPV, and it showed high sensitivity and specificity in accurately predicting movement disorders. These outcomes also resemble those of a study by Prodoehl et al. that found DTI can reliably identify and categorize individuals with essential tremors, atypical parkinsonism, and PD [17].

Previous research used different numbers of ROIs and voxels. One study by Hodaie et al. used a single ROI volume of 40 mm^3^, but the study by Vaillancourt DE et al. used four voxel diameters to define the ROI [10,16]. Three ROIs with a single voxel diameter were used in the current investigation. This study may have been better able to identify particular regions of degeneration in the SN and regional alterations because it chose to use three smaller ROIs based on the known degeneration pattern in PD. According to pathologic patterns of nigral degeneration, the caudal ROI was shown to be more impaired than the middle and rostral ROI. This shows that DTI-based FA is an anatomically focused assessment.

These results can be explained in two ways. First, the SN may not be the only object in the rostral region of the hypointense area of a T2WI. The SN and cerebral peduncle are located in the rostral portion of the hypointense region on an axial slice of a T2-weighted picture, according to T2WI combined with proton density-weighted, fast spin-echo STIR imaging. It is possible that the SN and cerebral peduncle were included in the rostral ROI of this study, which made it harder to distinguish between PD and healthy people in the rostral SN. However, as the most significant results were found in the caudal ROI, this constraint does not apply there.

The second theory is that patients with PD have more degeneration in the ventral and caudal regions of their SN than in other parts of the SN. The caudal ROI showed the most difference, which is in line with the area of the SN where dopaminergic cell loss in PD is most common. These findings align with the earlier research carried out by Reeve A et al. and Chan LL et al. [18,19]. Furthermore, the current discovery that the SN's caudal region is more damaged than its rostral segment is in line with recent proton density imaging methods that show a larger iron concentration in the caudal region, as demonstrated by the research done by Martin et al. [20].

In the past fifteen years, research has examined the use of MRI in parkinsonian illnesses, particularly in differentiating PD from atypical disorders like MSA and PSP. Although a number of morphological alterations have been identified as typical of MSA or PSP, the diagnostic sensitivity of these traditional MR indicators was generally low [21,22]. According to the current study, previous DTI/DTT studies have also demonstrated a higher sensitivity of this approach in the differential diagnosis of parkinsonian illnesses compared to traditional MR [23,24].

This study, like the one by Yoshikawa et al., found that individuals with late-stage MSA C had distinctive morphological abnormalities, particularly substantial pontine/cerebellar atrophy, pontine cruciform sign, and MCP signal modifications [21]. The DTI results demonstrated notable alterations in the MCP and TPF in advanced instances, including a noteworthy decline in the FA value in both the MCP and TPF, with the FA value in long-term disease decreasing more than in short-term disease. The FA of MCP and TPF both showed changes. Early illness stages showed a reduction in color, which was consistent with previously published research by Reimão S et al., Salamon et al., and Nicoletti et al. [12,25,26]. This suggested that DTI data might be crucial for differentiating between MSA from controls as well as from PSP and PD.

In this study, there was only one case of MSA P with long duration, which was also confirmed by MRI showing putaminal rim sign and basal ganglia atrophy. The DTI data showed changes in the MCP alone with MCP FA reduction. This reduction in FA was less compared to the FA value in MSA C similar to a study published by Reimão S et al. [12]. This study did not have any short-duration cases of MSA P. FA values were normal in SN, dentate nucleus, and SCP, and SCP fiber tract was normal in MSA C patients. FA values were normal in SN, dentate nucleus, SCP, TPF, and SCP, and TPF fiber tracts were normal in MSA P patients. In the present study, there was a mild reduction in TPF red color in the early stage of MSA C, and color reduction was also observed in MCP in MSA P patients, which was not seen in the previous study. These discrepancies could be due to interobserver variability in color interpretation and also because the duration of the disease was near the cut-off of long-duration disease. One patient in this study with MSA was diagnosed as normal by DTI, probably due to the use of a lower magnetic field strength MRI.

This study's findings, which are similar to those of Reimão S et al. and Nicoletti et al., showed that the patient with advanced disease had a decreased FA in the SCP and had lost the normal red color of the SCP decussation with relative integrity of the TPF and MCP [12,26]. These findings allowed for the patient to be differentially diagnosed with both PD and MSA cases in addition to controls. According to the Reimão S et al. study, there was also a little drop in FA in SCP and a decrease in the usual red color of the SCP decussation with relative integrity of the TPF and MCP in patients with early illness [12].

In PSP cases, FA value in the dentate nucleus was normal differentiating them from essential tremor patients. Only short-duration cases of essential tremors were present in this study. Five out of six patients were diagnosed with essential tremor by DTI. DTI showed FA value reduction in the dentate nucleus in these patients. FA value was also reduced in SCP but reduction was less compared to FA value reduction in PSP patients. The fiber tract was involved in SCP with a mild reduction of blue color. These findings were consistent with that of a previous study conducted by Nicoletti et al. [26]. One case of essential tremor was diagnosed as normal by DTI, probably due to use of lower magnetic field strength MRI. FA values were normal in MCP, TPF, and SN. MCP and TPF fiber tracts also were normal.

DTI in this study was able to subcategorize six atypical PD patients into four with PSP and two with MSA C. DTI was also able to subcategorize MSA into MSA C and MSA P and to diagnose 15 out of 17 short-duration cases of movement disorders, 28 out of 30 cases of movement disorders. These findings were significant and were done in a large number of patients compared to previous studies conducted by Vaillancourt DE et al. and Reimão S et al., done in small populations [10,12].

DTI measures the diffusion profile of water in the brain to obtain information on the tissue microstructure, which in turn can be used to estimate changes in white matter integrity and study the structure of cerebral tissue, such as the trajectories in white matter bundles and the orientation fibers [27,28]. This helps in detecting even slight changes in the brain happening during degenerative diseases making DTI more sensitive in diagnosing them at an early stage. DTI can thus help in the early management of patients suffering from movement disorders as well as in understanding the pathophysiology of these disorders at the earliest stages.

## Conclusions

DTI has high sensitivity as well as specificity for diagnosis of movement disorders. It is capable of differentiating between various types of movement disorders. DTI also plays a significant role in the early diagnosis of movement disorders compared to conventional MR imaging. Futuristically, DTI protocol can be included in all patients undergoing MRI brain for movement disorder. There is a need to conduct longitudinal studies using DTI on larger cohorts of patients with parkinsonian syndromes, particularly including at-risk and early disease populations so that these preliminary findings can be investigated. Future research can be facilitated by the increasing availability of higher field and multimodal neuroimaging and would benefit from greater congruity in MRI protocols.
